# Immunosuppressive tumor microenvironment and immunotherapy of hepatocellular carcinoma: current status and prospectives

**DOI:** 10.1186/s13045-024-01549-2

**Published:** 2024-04-29

**Authors:** Ke-Yu Shen, Ying Zhu, Sun-Zhe Xie, Lun-Xiu Qin

**Affiliations:** 1 Hepatobiliary Surgery, Department of General Surgery, Huashan Hospital & Cancer Metastasis Institute, Fudan University, 12 Urumqi Road (M), Shanghai, 200040 China; 2 Institutes of Biomedical Sciences, Fudan University, Shanghai, 200032 China

**Keywords:** Hepatocellular carcinoma, Tumor microenvironment, Immune checkpoint inhibitors, Programmed death receptor 1, Adoptive cell therapy, Neoantigen vaccine, Oncolytic virus, Bispecific antibody

## Abstract

Hepatocellular carcinoma (HCC) is a major health concern worldwide, with limited therapeutic options and poor prognosis. In recent years, immunotherapies such as immune checkpoint inhibitors (ICIs) have made great progress in the systemic treatment of HCC. The combination treatments based on ICIs have been the major trend in this area. Recently, dual immune checkpoint blockade with durvalumab plus tremelimumab has also emerged as an effective treatment for advanced HCC. However, the majority of HCC patients obtain limited benefits. Understanding the immunological rationale and exploring novel ways to improve the efficacy of immunotherapy has drawn much attention. In this review, we summarize the latest progress in this area, the ongoing clinical trials of immune-based combination therapies, as well as novel immunotherapy strategies such as chimeric antigen receptor T cells, personalized neoantigen vaccines, oncolytic viruses, and bispecific antibodies.

## Introduction

Hepatocellular carcinoma (HCC) is a major health concern worldwide, and is the third leading cause of cancer-related death globally [1, 2]. HCC frequently presents as an advanced disease at diagnosis, and disease relapse following surgical treatments is frequent, making systemic therapy an important part of treatment [3]. Recently, immunotherapies, particularly immune checkpoint inhibitors (ICIs), have demonstrated impressive clinical benefits in various solid tumors including HCC [4].

Immunotherapy essentially enhances cellular immunity, regulates the degree of immune activation, and improves T-cell function, thereby inhibiting the occurrence and growth of tumor cells. Immunotherapeutic approaches for HCC include ICIs, adoptive cell therapy (ACT), oncolytic virotherapy, and cancer vaccine therapy, etc. Both monotherapy and the combinations with other treatments have obtained much progress recently, which has brought a change in the therapeutic paradigms of HCC.

In this review, we summarize the latest advances in understanding the immunosuppressive characteristics of tumor microenvironment (TME), established immunotherapy strategies such as ICIs, as well as some emerging strategies of immunotherapy for HCC.

## Immunosuppressive microenvironment of HCC

**Fig. 1:**
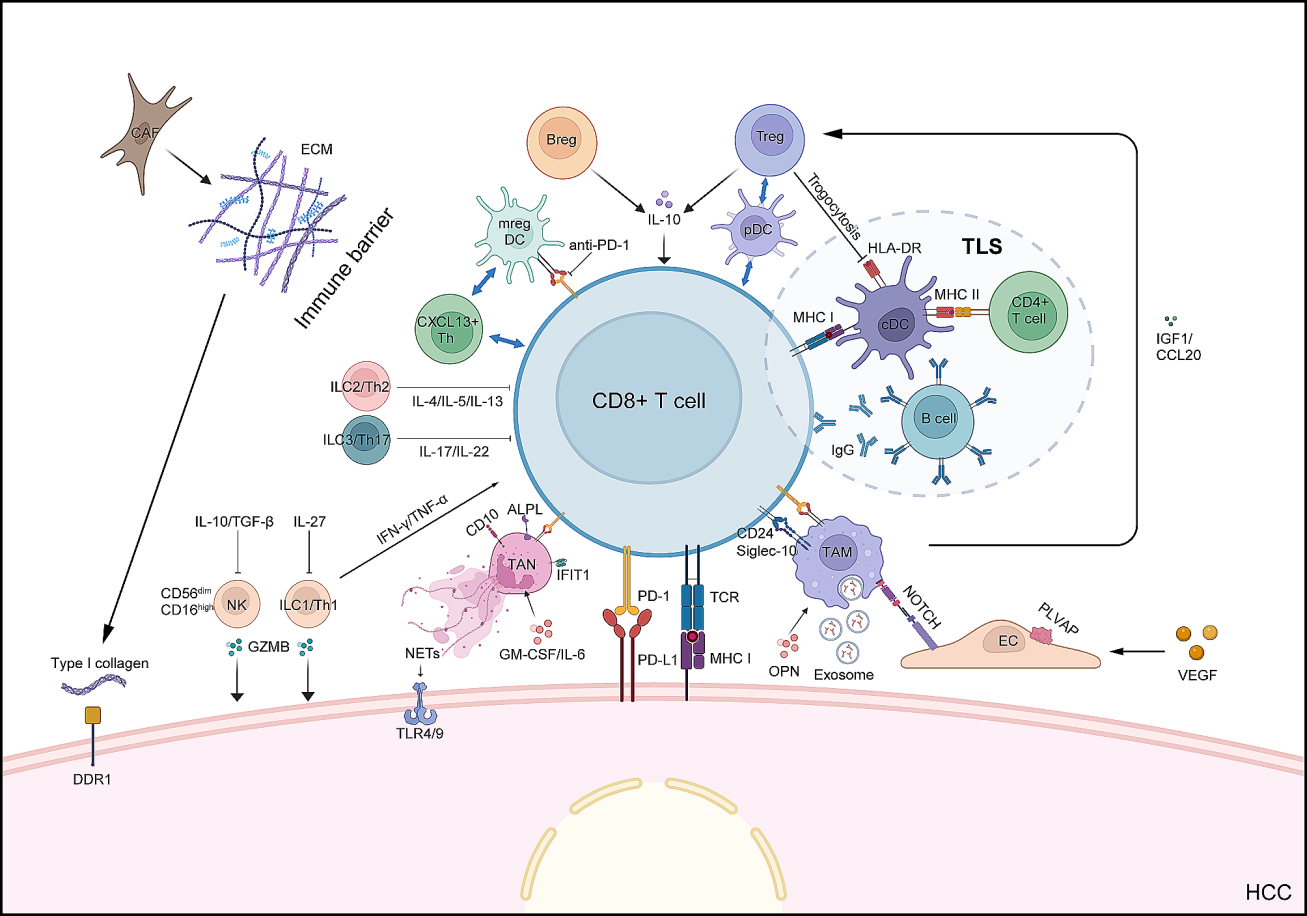
Immune microenvironment of hepatocellular carcinoma. Multiple cell types, cytokines, and other components contribute to the heterogeneous immune microenvironment. Tumor cells instruct neutrophils and macrophages to become TAMs and TANs through exosomes, secreted proteins (such as OPN), and cytokines (such as IL-6), thereby suppressing T cell function. cDCs interact with B cells and T cells, forming TLS, which facilitates antigen presentation and activation of T cells. Immunosuppressive cells such as mregDCs, Tregs, and Bregs inhibit anti-tumor immunity of T cells through direct contact with immune checkpoints or secretion of IL-10. ILCs are a heterogeneous population, in which NK cells and ILC1s possess cytotoxicity that can directly destroy tumor cells while ILC2s and ILC3s mirror TH2 and TH17 cells, respectively, which suppress immune responses by secreting cytokines like IL-13 and IL-17. CAF builds a tumor immune barrier which hinders the infiltration of immune cells through the secretion of ECM. Tumor-associated ECs promote monocyte differentiation into TAMs through the NOTCH signaling. Figures were created with BioRender. Abbreviations: CAF, cancer-associated fibroblast; ECM, extracellular matrix; DDR1, discoidin domain receptor 1; IL, interleukin; TGF-β, transforming growth factor-beta; NK, natural killer; ILC, innate lymphoid cell; Th, helper T cell; GZMB, granzyme B; Breg, regulatory B cell; mregDC, mature dendritic cell enriched in immunoregulatory molecules; TAN, tumor associated neutrophil; IFN, interferon; TNF-α, tumor necrosis factor-α; NET, neutrophil extracellular trap; TLR, Toll-like receptor; Treg, regulatory T cell; pDC, plasmacytoid dendritic cell; cDC, conventional dendritic cell; TLS, tertiary lymphoid structures; MHC, major histocompatibility complex; IGF, insulin-like growth factor; CCL, C-C motif ligand; TAM, tumor-associated macrophage; PD-1, programmed death receptor 1; PD-L1, programmed death-ligand 1; OPN, osteopontin; EC, endothelial cell; HCC, hepatocellular carcinoma

The liver possesses intact immune features, which together build a strong immune system network for HCC under its strong immunogenicity as well as microbial environment [4]. In addition, HCC occurrence is associated with infection and inflammation, in which hepatitis B virus (HBV) or hepatitis C virus (HCV) may reduce T cell activity and mediate immunosuppression through the programmed death receptor 1 (PD-1) pathway. The TME is composed of various cell types, cytokines, and other components. The cytotoxic immune responses in the HCC tumor microenvironment are blunted, as shown by the enrichment of tumor-promoting immunosuppressive cell types including lymphocytes and myeloid cells [5, 6]. These cells further interact with neutrophils, dendritic cells (DCs), B cells, and others, leading to impaired innate and adaptive immunity against HCC [7] (Fig. 1; Table 1). Understanding the mechanisms through which these immune cells contribute to tumor immune evasion is of paramount importance in guiding immunotherapy.

**Table 1:** Properties of tumor microenvironment cells in hepatocellular carcinoma

Cell type	Markers	Properties in HCC	Reference
TAMs	OPN, C1QA, THBS1, APOE, SLC40A1, GPNMB	Related with reduced CD8 + T cell infiltration in TME and poor prognosis.	[5, 10]
TANs	MMP8, APOA2, CD74, IFIT1, SPP1, CCL4	Inhibited the cytotoxicity of CD8 + T cells by elevating PD-L1 expression.	[32]
cDCs	CLEC9A, XCR1, CADM1(cDC1); CD1C, FCER1A, CLEC10A (cDC2)	Primarily responsible for antigen presentation.	[10]
pDCs	BDCA2, ILT7	Associated with infiltration of Tregs and poor prognosis of HCCs.	[38]
Migratory DCs	LAMP3, CD80, CD83, CCR7, CCL19, CCL21	Migratory DCs exhibited migratory capacity and a strong correlation with TEX.	[10]
NK cells	CD56, CD16	The higher number of NK cells correlated with a positive prognosis in HCC.	[50]
Helper ILCs	CD127	Functional heterogeneity.	[51]
Activated CD8 + T cells	CD8, GZMB	Associated with a favorable prognosis in HCC.	[66]
TEX	CD8, PD-1, TIM-3	Enrichment of TEX was linked to poor PFS and OS.	[5, 64]
TRM	CD69, CD103	Associated with a better prognosis and response to immunotherapy.	[64]
Tregs	CD4, FOXP3, CTLA-4	Tregs mediate T cell exhaustion and are associated with poor prognosis.	[5, 66]
Th	IFN-γ, IL-2 (Th1); IL-4, IL-5 (Th2); IL-17 (Th17)	Different Th subtypes exert positive or negative effects on the immune response.	[75]
B cells	CD19, CD20	Main constituent of TLS which correlates with a favorable prognosis.	[84]
CAFs	α-SMA, COL1A2, COL1A1	Mediated immune evasion by direct interactions, secretion of cytokines, and ECM.	[97]
ECs	PLPP3, IGFBP3, PLVAP	Interacted with TAMs and CAFs to attenuate the response of immunotherapy.	[52]

TAM, tumor-associated macrophage; TAN, tumor associated neutrophil; cDC, conventional dendritic cell; pDC, plasmacytoid dendritic cell; NK, natural killer; ILC, innate lymphoid cell; TEX, exhaustion T cell; TRM, tissue-resident memory T cell; Treg, regulatory T cell; Th, helper T cell; CAF, cancer-associated fibroblast; EC, endothelial cell; TME, tumor microenvironment; PD-L1, programmed death-ligand 1; HCC, hepatocellular carcinoma; PFS, progression-free survival; OS, overall survival; TLS, tertiary lymphoid structures; ECM, extracellular matrix

### Tumor-associated macrophages (TAMs)

Macrophages are the most abundant innate immune cells in TME [8–10]. Two subtypes of TAMs are identified, the M1 subtype (classically activated macrophages) and the M2 subtype (alternatively activated macrophages). M1/M2 dichotomy is based on the different functions in tumor progression. Immunoregulator molecules, such as IL-4, IL-10, macrophage colony-stimulating factor (M-CSF), and transforming growth factor-beta (TGF-β), could induce M2 polarization, while pro-inflammatory cytokines, like IL-1β, IL-6, IL-12, and tumor necrosis factor-α (TNF-α), trigger M1 phenotype [11].

TAMs facilitate HCC progression through multiple pathways including angiogenesis, cancer cell reshaping, immunosuppression, extracellular remodeling, and drug resistance [12]. TAMs induce immunosuppression through expressing inhibitory immune checkpoints such as PD-1, programmed death-ligand 1 (PD-L1), and T cell immunoglobulin and mucin domain-3 (TIM-3); secreting immunosuppressive cytokines such as IL-6; and recruiting immunosuppressive cells including regulatory T cells (Tregs) [13–15]. In addition, TAMs with a high sialic-acid-binding Ig-like lectin 10 (Siglec-10, an inhibitory receptor of immune checkpoint CD24) (Siglec-10 + TAMs) were demonstrated to help tumor cells to escape from immunosurveillance and phagocytosis [16]. Some TAMs-secreted proteins, like S100 calcium-binding protein A9 (S100A9), could elevate the cancer stem cell-like ratio in HCC [17]. Meanwhile, TAMs are important meditators between tumor cells and other immune effector cells. M2 TAMs secreted insulin-like growth factor-1 (IGF-1) and C-C motif ligand 20 (CCL20) to recruit Tregs and impair CD8 + T cell effects [18]. Our previous study revealed that GLOM1, which was upregulated in metastatic HCC cells, could promote PD-L1 transportation from HCC cells to TAMs through exosomes. The increased PD-L1 + TAMs suppressed CD8 + T cell functions and fueled HCC progression and metastasis [19]. Osteopontin (OPN), an important pro-metastasis gene, could promote macrophage infiltration and PD-L1 expression in HCC through stimulating colony stimulating factor 1/ colony stimulating factor 1 receptor (CSF1/CSF1R) pathway in macrophages. Targeting macrophages using CSF1R inhibitor significantly improved CD8 + T cell infiltration and enhanced the efficacy of PD-L1 antibody [20].

Many novel subtypes of macrophages have been identified in HCC. Using single-cell RNA sequencing (scRNA-seq), two main subtypes of macrophages, C1QA + and THBS1 + macrophages, were demonstrated to be accumulated in HCC tissues. C1QA + macrophage was defined as TAM-like macrophage, with M1 and M2 signatures and high expression of APOE, SLC40A1, and GPNMB; THBS1 + macrophage was myeloid-derived suppressor cell (MDSC)-like. TAM-like macrophage was closely associated with poor prognosis of HCC [6]. Ho et al. found that TAMs accumulation, especially LAIR1 + and TIM3 + macrophages, was related to reduced CD8 + T cells infiltration and poor prognosis in HCC [10]. Hao et al. showed that APOC1 + macrophage was abundant in HCC tissues, and APOC1 inhibitor could reshape M2 macrophage to M1 phenotype and enhance the anti-PD-1 immunotherapy [21]. The complex function of TAMs spurs much interest in developing new therapeutic strategies targeting macrophages. The combination of CCR2/CCR5 antagonist (targeting macrophage) with Nivolumab is now in a phase II study (NCT04123379).

### Tumor-associated neutrophils (TANs)

Neutrophil is the fastest cell type to arrive at lesion sites and initiate innate immunity to defend against pathogen invasion, eliminate damaged tissues as well as shape the adaptive immune response. TANs can be divided into N1, N2, and polymorphonuclear myeloid-derived suppressor cells (PMN-MDSCs) mainly based on their functions and markers. N1 is believed to be equipped with anti-tumor effects, while N2 and PMN-MDSC have pro-tumor functions through immunosuppression, angiogenesis, or direct enhancing aggressive characteristics of tumor cells [22, 23]. HCC cells or other stromal cells in TME could secret cytokines, chemokines, or inflammatory molecules to influence neutrophil mobilization, recruitment, and polarization. Some molecules, including granulocyte-macrophage colony-stimulating factor (GM-CSF), IL-6, TGF-β and prostaglandin E2 (PGE2), can educate neutrophil to polarize toward pro-tumor phenotype [24]. High neutrophil-lymphocyte ratio (NLR) is used as a marker of the dismal prognosis of HCC [25].

Another widely studied topic is NETosis, a process of forming neutrophil extracellular traps (NETs) [26]. NETs, a structure containing chromatin, histone protein, and intracellular protein, are produced from a unique neutrophil death and were once recognized as a powerful weapon against pathogens [27]. However, NETs are proved to facilitate the progression and metastasis of many cancers including HCC [28–30]. In our study, we found that NETs triggered tumorous inflammatory responses and fueled HCC metastasis. We also proposed the combination of DNase I (directly wrecked NETs) with aspirin (an anti-inflammation drug) effectively reduced HCC metastasis in mice models [31].

The heterogeneity of neutrophils makes them have pro- or anti-tumor effects. However, considering the complex functions of neutrophils in tumor progression, classification simply based on bulk RNA sequence seems non-comprehensive sometimes. Due to the low RNA abundance and short lifespan, neutrophils were once too difficult to be captured and identified by scRNA-seq until the applications of the optimized workflow (like enrichment-free strategy [32]) or capture methods (like BD Rhapsody platform [33]). In HCC, using scRNA-seq, 11 subsets of neutrophils were identified, which exhibited distinct transcriptomic and functional signatures. Among them, neutrophils with high expression of MMP8, APOA2, CD74, IFIT1, SPP1, or CCL4 were accumulated in HCC and defined as TANs. TANs elevated PD-L1 expression and fueled tumor growth by inhibiting the cytotoxic function of CD8 + T cells. Meng et al. revealed CD10 + ALPL + neutrophils mediated resistance to anti-PD-1 treatment through irreversibly exhausting T cells in HCC [34]. However, Sell^high^ neutrophils dramatically accumulated in the lung cancers of successful immunotherapy and the systemic neutrophil response is positively related to the patients’ outcomes [35]. In melanoma, T cell immunotherapies also recruited and activated neutrophils to eliminate antigen escape variants of tumor cells through NETosis and iNOS production, highlighting the important role of neutrophils in complete tumor eradication [36]. These indicate that some specific neutrophil subsets appear to be essential for successful immunotherapy. Many clinical studies about the combination of neutrophil-targeted therapy with other immunotherapies are undergoing in HCC (NCT02423343, NCT03785210) and other cancers (NCT02583477, NCT03990233). Unfortunately, the specific role of neutrophils in immunotherapy remains obscure.

### Dendritic cells

DCs, as antigen-presenting cells (APCs), interact with various immune cells such as CD4 + and CD8 + T cells, serving as a bridge between innate and adaptive immunity and playing a crucial role in TME. DCs are commonly divided into conventional DCs (cDCs), including cDC1 and cDC2 and plasmacytoid DCs (pDCs). cDCs are primarily responsible for antigen presentation, while pDCs mainly participate in antiviral and antitumor immunity through the production of type I interferon [37].

The immune response to immunotherapy largely relies on DC cells, however, DC cells often exert immunosuppressive effects in HCC. High density of tumor-infiltrating pDCs was associated with infiltration of Tregs and poor prognosis of HCCs [38–40]. Tregs can inhibit the expression of HLA-DR on cDC2s through a mechanism similar to trogocytosis [41], in which one cell physically extracts and ingests cellular material from another cell, thereby impairing its antigen presentation function [42].

Recently, scRNA-seq has revealed the heterogeneity of DC cells in HCC and further accurately identified the subsets of DC cells and their functions. A novel subset of mature DCs, CCR7 + LAMP3 + DCs were identified to exhibit a migratory capacity from tumors to lymph nodes and a strong correlation with exhaustion T cells (TEX) and Tregs, suggesting their involvement in T cell dysfunction [6]. They were also found in lung cancer, and were named as mature dendritic cells enriched in immunoregulatory molecules (mregDCs) due to their co-expression of immunoregulatory markers (Cd274, Pdcd1lg2, and Cd200) and maturation markers (Cd40, Ccr7, and Il12b) [43]. In the context of ICI therapy, there is a significant enrichment of cellular triads composed of mregDCs, CXCL13 + helper T (Th) cells, and PD-1^hi^ progenitor CD8 + T cells within the HCC microenvironment. The communication between mregDC and CXCL13 + Th cells within these cellular triads assists the progenitor CD8 + T cells to differentiate into effector anti-tumor CD8 + T cells [44]. Similarly, CXCR3 + CD8 + effector memory T (TEM) cells and HLA-DR + cDC1s that are recruited into the TME determine the responsiveness of HCC to ICI [45]. Given the crucial role of DCs in tumor immune responses, many DC-based treatment strategies, including DC vaccines, and the combination therapy of DCs with cytokine-induced killer cells (DC-CIK), have been employed in the clinical management of HCC [46]. In preclinical studies, using nanodrugs to stimulate DC cells [47, 48] or employing DC-derived exosomes [49] have been demonstrated to activate tumor-specific immunity against HCC, which represents a potential direction for the future development of immunotherapy.

### Innate lymphoid cells

Innate lymphoid cells (ILCs) are a highly heterogeneous family including circulating natural killer (NK) cells, ILC1s, ILC2s, and ILC3s. According to their functions and cytokine secretion profiles, NK cells are considered the innate counterparts of CD8 + T cells, while ILC1s, ILC2s, and ILC3s mirror the functions and characteristics of CD4 + helper T cell subsets. ILC1s secrete interferon-γ (IFN-γ) and TNF-α, which are the characteristics of Th1 cells. ILC2s secrete IL-4, IL-5, and IL-13, which are typically associated with Th2 cells. ILC3s secrete IL-17 and IL-22, which are the signature of Th17 cells [50, 51].

NK cells are the major lymphocyte population in the human liver, accounting for approximately 50% of the total intrahepatic lymphocytes [52]. They can be divided into two subsets: cytotoxic CD56^dim^CD16^high^ NK cells and immunomodulatory CD56^bright^CD16^low^ NK cells [50]. IL-10 increases the expression of NKG2A [53], and TGF-β upregulates CD96 [54], both leading to the exhausted phenotype of CD11b-/CD27- NK cells [55]. Targeting IL-10 or TGF-β can reverse the dysfunction of NK cells. The accumulation of lactate leads to a significant decrease of nicotinamide adenine dinucleotide (NAD+) in NK cells, resulting in their dysfunction. Supplementation with the NAD + precursor nicotinamide mononucleotide (NMN) significantly improves the anti-tumor effects of NK cells [56]. Owing to the potent cytotoxicity of NK cells against tumor cells, various NK cell-based therapeutic strategies have been explored in HCC, including the use of bispecific antibodies to enhance antibody-dependent cellular cytotoxicity (ADCC) effect, as well as the adoptive transfer of CIK cells and chimeric antigen receptor (CAR)-engineered NK cells [57].

ILC1s recently tend to be classified as distinct cell types from NK cells, their role in cancer is still controversial. The heterogeneity of ILC1s may be attributed to the presence of distinct subsets (cytotoxic and noncytotoxic) and their proportions within ILC1s population [58, 59]. In HCC, blockade of IL27/IL27R axis was shown to generate “cytotoxic-like” ILC1 populations [60].

Similar to ILC1s, ILC2s also exhibit a dual role in HCC. Inducible T-cell co-stimulator (ICOS) + ILC2a cells were enriched in HCC and associated with poor prognosis. The inducible T-cell co-stimulator ligand (ICOSL) signaling derived from the resting naive B cells increased the secretion of IL-13 by ICOS + ILC2a cells to promote HCC development [61]. Another study revealed that under the influence of IL-33, ILC1s, and ILC3s can undergo plasticity and transform into activated ILC2s, and a high ILC2/ILC1 ratio is associated with enhanced anti-tumor immune responses and better prognosis [62]. IL-23 can promote the expansion of natural cytotoxicity-triggering receptor (NCR) negative ILC3 and induce the secretion of IL-17, leading to an immunosuppressive tumor microenvironment in HCC [63]. However, understanding of ILCs (aside from NK cells) is still relatively limited, and further studies are needed to develop effective ILCs-targeting strategies to combat HCC.

### T cells

As the major cell type that is targeted by ICI therapy, CD4 + T cells and CD8 + T cells often exhibit a dysfunctional or exhausted phenotype in HCC. Single-cell analyses have revealed significant infiltration of Tregs and exhausted CD8 + T cells (TEX) in HCC [5]. Large-scale clinical sample studies have demonstrated their negative correlation with the prognosis and response to immunotherapy of HCC [64–66]. TEX cells are characterized by significant transcriptional changes, such as upregulation of thymocyte selection associated high mobility group box (TOX), and the expression of a range of inhibitory receptors, including PD-1, cytotoxic T-lymphocyte antigen 4 (CTLA-4) and lymphocyte activation gene 3 (LAG3) [67, 68]. Previously, it was believed that T cell exhaustion was caused by long-term continuous antigen stimulation and exposure to an immunosuppressive TME. However, a recent study found that T cells can begin to exhibit exhaustion within a few hours of tumor antigen encounter [69]. Tissue-resident memory CD8 + T (TRM) cells, as a distinct type of memory T cells, are characterized by the presence of CD69 and CD103 markers [70]. TRM cells can also express PD-1 and are enriched in tumors, particularly in HBV-related HCC. In contrast to exhausted T cells, non-terminally exhausted TRM cells are associated with a better prognosis and response to ICI therapy, suggesting TRM cells may be novel targets for immunotherapy [64, 71, 72].

On the other hand, CD4 + T cells also attract wide interest due to their double-edged effects in HCC progression and ICI therapy. Naïve CD4 + T cells proliferated and differentiated into Th cells, T follicular helper (Tfh) cells, and Treg cells under co-stimulation of activated APCs and cytokines [73]. Th cells differentiated into Th1, Th2, and Th17 depending on variant cytokines derived from other innate immune cells. Th1 cells promote anti-tumor effects of CD8 + T cells by secreting IFN-γ and IL-2 and accelerate DC maturation through the CD40-CD40L axis [74, 75]. Th17 was abundant in HCC and associated with poor prognosis and microvessel density [76, 77]. Meanwhile, Th17 increases the PD-L1 expression level in HCC cells through secreting IL-17 A, resulting in resistance to PD-L1 treatment [78].

Tregs display immune-suppressive characteristics and impair the effects of APCs by highly expressing CTLA-4, which competitively binds to CD80/CD86 on APCs [79]. The stem-like CCR4 + Tregs were revealed to play a major immunosuppressive role, particularly in HBV-related HCC. Blocking CCR4 can enhance the efficacy of ICIs [80]. Interestingly, although Tregs are enriched in primary HCC, early-relapse HCC displays significant heterogeneity, characterized by decreased Tregs, increased CD8 + T cells, and DCs. These CD8 + T cells overexpress CD161 and exist in an innate-like low cytotoxic state, suggesting that they are unable to mount effective immune responses against neoantigens, leading to immune evasion [81]. Several studies have demonstrated that Treg exerts a crucial role in ICB therapy. The high Treg infiltration is related to reduced clinical benefits of anti-PD-L1 plus anti-VEGFR treatment [82]. However, given the crucial role of Tregs in auto-immune diseases, further exploration of precisely inhibiting tumor-infiltrating Tregs through ICI therapy without compromising the physiological function of Tregs is a worthwhile endeavor.

### B cells

Increasing evidence indicates that tumor-infiltrating B cells (TIBs) and plasma cells (PCs) play pivotal roles in tumor immunity by participating in antigen presentation, antibody production, cytokine secretion, and other functions [83]. The presence of TIBs and PCs is correlated with a favorable prognosis of HCC [84, 85]. The most recent study on tertiary lymphoid structures (TLS) provided additional support for the involvement of B cells in tumor immunity. B cells serve as the main constituent of TLS and undergo transformation into PCs within TLS and produce IgG antibodies to combat tumors [86]. The occurrence of TLS in the vicinity of tumors is correlated with a positive prognosis of cancers [87, 88]. However, Calderaro et al. found that the presence of intra-tumoral TLS, but not non-tumoral TLS, was associated with a reduced risk of early recurrence in 273 patients who underwent surgical resection for HCC [89]. In addition, the occurrence of immature TLS that lacks DCs and efficient immune reactions in preneoplastic/early hepatic lesions is correlated with stronger immunosuppression and T cell exhaustion [90]. The underlying causes for these discrepancies could be the etiology (HBV or HCV infection), stage (early or advanced) of HCC, as well as the localization of TLS (within or outside HCC). Thus, the precise role of TLS in HCC and the development of effective intervention strategies require further investigation.

Many studies indicate that B cells that produce IgA antibodies can also play a role in promoting liver fibrosis and HCC development, particularly in non-alcoholic steatohepatitis (NASH)-driven HCC [91]. IgA + B cells upregulate the expression of PD-L1 and inhibit the activation and cytotoxicity of T cells by secreting IL-10, and genetic or therapeutic depletion of IgA + B cells is beneficial in alleviating liver fibrosis [92] and reducing liver carcinogenesis [93]. Regulatory B cells (Bregs), as a novel subset of B cells characterized by their secretion of IL-10, not only inhibit T cell function but also directly promote the progression and vascular invasion of HCC through the CD40/CD154 axis [94]. Very recently, it was worth noting that blocking Ten-eleven translocation-2 (TET2) can restrain IL-10-producing Bregs and enable antitumor immunity [95].

### Non-hematopoietic stromal cells

In HCC, other stromal cells can also interact with immune cells and ultimately contribute to immunosuppression TME. The accumulation of cancer-associated fibroblasts (CAFs) is a prominent feature of HCC, which mediates immune evasion through receptor-ligand interactions, secretion of various cytokines, and remodeling of the extracellular matrix (ECM) [96]. CAFs in HCC are a heterogeneous population that primarily originates from hepatic stellate cells (HSCs) and can be classified into five subgroups by scRNA-seq. These subgroups include vascular CAFs (vCAFs), which express microvasculature genes; antigen-presenting CAFs (apCAFs), which express MHC II genes; matrix CAFs (mCAFs), which express extracellular matrix (ECM) signatures; lipid-processing CAFs (lpCAFs), which express lipid metabolism-related genes; and CD36 + lipid processing mCAFs (lpmCAFs), which are enriched in both ECM formation and lipid metabolism and specifically abundant in HCC. CD36 + lpmCAFs can recruit CD33 + MDSCs through a macrophage migration inhibitory factor (MIF)-dependent pathway, thereby mediating tumor immune evasion. Therefore, CD36 inhibitors synergistically enhanced T-cell responses in combination with anti-PD-1 therapy [97]. CAFs can also promote the secretion of CXCL6 and TGF-β in HCC cells by producing cardiotrophin-like cytokine factor 1 (CLCF1), which indirectly facilitates the “N2” polarization and infiltration of TANs [98]. Moreover, recent spatial transcriptomic analysis has revealed the direct interaction between CAFs and SPP1 + macrophages through a receptor-ligand network, thereby remodeling the ECM and forming a tumor immune barrier around HCC, which hinders the infiltration of immune cells [99]. Similarly, our recent research has revealed the presence of “a pro-tumor cirrhotic-ECM” in HCC, featured by upregulation of type I collagen, which impaired the function of T cells and limited the response of patients to anti-PD-1 therapy. Mechanically, type I collagen recruited neutrophils to promote NETs formation through discoidin domain receptor 1 (DDR1)-CXCL8 axis, thereby shielding tumor cells from T-cell attack. DDR1 inhibitors can reverse the formation of NETs and enhance the efficacy of anti-PD-1 therapy [100].

Tumor vascular endothelial cells (ECs) are one of the key stromal cells in the TME that participate in angiogenesis and tumor metastasis, as well as in modulating immune responses and the efficacy of anti-cancer drugs. Liver sinusoidal ECs (LSECs) are a unique type of ECs in the healthy liver, characterized by the discontinuous basement membrane. However, during hepatocarcinogenesis, discontinuous LSECs are gradually replaced by continuous ECs, which is critical for HCC angiogenesis [101]. The scRNA-seq of HCC identified 11 distinct EC clusters, of which PLPP3+, IGFBP3+, and PLVAP + ECs were found to be enriched in the tumor tissue. Interestingly, PLVAP + ECs were identified to be HCC-specific and only present in fetal liver, but not in healthy liver, suggesting their involvement in onco-fetal reprogramming of HCC. Hepatocyte-derived vascular endothelial growth factor (VEGF) signaling promotes the expression of PLVAP in ECs, which in turn induces the differentiation of monocytes into fetal-like FOLR2 + TAMs through NOTCH signaling [52]. The co-localization and interaction of POSTN + ECM-secreting CAFs, FOLR2 + TAMs, and PLVAP + ECs promote the progression of HCC and recruit Tregs to attenuate the response to immunotherapy [102]. Furthermore, the post-transarterial chemoembolization (TACE) TME exhibits a significant enrichment of TREM2 + TAMs, which secrete Galectin-1 to promote the expression of PD-L1 in ECs [103]. These findings provide a strong rationale for the synergistic use of anti-angiogenic drugs and anti-PD-1/PD-L1 therapy in the treatment of HCC.

### Platelets

Platelets are anucleate cytoplasmic vesicles produced by megakaryocytes. In addition to their physiological function in hemostasis, increasing evidence suggests their crucial role in the development and progression of HCC [104]. The adhesion and activation of platelets in the liver have been shown to promote the progression of NASH and subsequent formation of HCC in mice. This may be attributed to the elevated levels of chemokines and cytokines after activation of platelets [105]. In a chronic hepatitis B mouse model, anti-platelet therapy effectively prevented the formation of HCC [106]. Clinically, chronic hepatitis patients who received long-term treatment with the anti-platelet medication aspirin exhibited a lower risk of developing HCC [107]. Surprisingly, after the formation of HCC, platelets paradoxically inhibit the progression of HCC as the platelet-secreted CD40L activates the anti-tumor efficacy of CD8 + T cells [108]. Moreover, after insufficient radiofrequency ablation (RFA) for HCC, the upregulation of ICAM-1 in tumor ECs triggers platelet aggregation and activation, leading to increased endothelial permeability through VE-cadherin, and facilitating tumor transendothelial migration. Treatment with anti-platelet or anti-ICAM-1 agents after RFA may potentially prevent HCC recurrence and metastasis [109].

### The heterogeneity of TME and response to immunotherapy

The heterogeneity of the immune microenvironment in HCC makes it increasingly complex to predict prognosis and guide clinical treatment. Some studies have attempted to integrate multi-omics technologies (genomics, transcriptomics, proteomics, etc.) and clinical prognosis in order to classify HCC based on immunology [110–112]. Based on bulk sequencing data, using the markers of inflammatory response (PD-1, PD-L1) and cytolytic activity, Sia et al. classified approximately 25% of HCC cases as immune class, which was further divided into two subtypes, immune-activated (characterized by overexpression of adaptive immune response genes and better prognosis) and immune-exhausted (characterized by TGF-β expression, enrichment of M2 macrophages, and T cell exhaustion) [110]. By integrating analysis of single-cell and bulk data, Zhang et al. categorized HCC into three subtypes: immunodeficient, immunocompetent, and immunosuppressive. The immunodeficient subtype, similar to “cold” tumors, is characterized by reduced infiltration of lymphocytes, which limits the effectiveness of ICIs. Therefore, the combination of ICIs with tyrosine kinase inhibitors (TKIs), oncolytic viruses (OVs), or other approaches that increase lymphocyte infiltration may be applicable to this subtype. The immunocompetent subtype, consistent with the previously mentioned immune-activated subtype, exhibits normal T cell infiltration and is associated with a favorable prognosis. Combining ICIs with T cell stimulators such as IL-12 may further enhance anti-tumor immunity. The immunosuppressive subtype, resembling the immune-exhausted subtype, is characterized by infiltration of immunosuppressive cells (including Tregs, Bregs, and M2 macrophages) and upregulation of immune checkpoints (including PD-1, PD-L1, and TIM-3). In this situation, monotherapy with ICIs can sustain or reverse the exhaustion of T cells [111]. Although these immune subtypes can provide valuable insights into treatment strategies, it is important to note that they have not been validated in clinical cohorts. Therefore, additional clinical trials are necessary to establish the clinical relevance and applicability of this classification.

## Clinically established immunotherapies

### ICIs

Immune checkpoints are a series of regulatory molecules expressed by immune cells that play an important role in maintaining autoimmune function and autochthonous diseases, which includes the PD-1 and its ligands (PD-L1/PD-L2), mucin molecules, lymphocyte activation genes, CTLA-4, and lymphocyte attenuation factors. PD-1/PD-L1 and CTLA-4 are the most widely used targets for HCC in immunotherapy, mainly focusing on PD-1/PD-L1 mono-antibodies, which have become world-class “stars”.

Since the pilot phase II trial of the CTLA-4 blockade tremelimumab as a single agent in 21 patients with advanced HCV-associated HCC with an objective response rate (ORR) of 17.6% [113], PD-1 antibodies, nivolumab and pembrolizumab had been approved by U.S. Food and Drug Administration (FDA) as the 2nd line treatment for advanced HCC based on the results of the CheckMate 040 and KEYNOTE-224 trials, in which nivolumab and pembrolizumab produced an ORR of 15–20% including 1–5% complete response (CR), and prolonged survivals [114, 115]. However, the confirmatory phase III trials of nivolumab (CheckMate 459) and pembrolizumab (KEYNOTE-240) alone as the first-line and second-line settings, respectively, failed to reach the predefined statistical significance [116, 117], even though they showed impressive anti-tumor activity with an ORR of 14-17% and durations of response > 12 months. FDA opposed the maintenance of accelerated approval of nivolumab monotherapy for advanced HCC patients previously treated with sorafenib. Therefore, the indication of nivolumab as post-sorafenib monotherapy for advanced HCC was withdrawn. But in patients from Asia with previously treated advanced HCC, pembrolizumab significantly prolonged overall survival (OS) (14.6 vs. 13.0 months; HR 0.79; 95% CI 0.63 to 0.99; *p* = 0.0180), progression-free survival (PFS) (2.6 vs. 2.3 months; HR 0.74; 95% CI 0.60 to 0.92; *p* = 0.0032), and ORR (12.7% vs. 1.3%; *p* < 0.0001) versus placebo [118]. In addition to pembrolizumab and nivolumab, more and more PD-1 monoclonal antibodies are available on the market. Overall, ICI monotherapy showed an encouraging 15–20% ORR and a good safety profile but failed to show a statistically significant advantage over established TKI treatments, leading researchers to explore combination strategies in efforts to improve efficacy. Several novel combinations including anti-PD-1/PD-L1 antibodies in combination with anti-VEGF antibodies, TKIs, CTLA-4 inhibitors, or other regional therapies have been developed [119–122] (Table 2).

**Table 2:** Randomized phase III trials of ICI-based combination therapy in advanced hepatocellular carcinoma

Study	Drug	Start	*N*	mOS	mPFS
IMbrave150 [120]	Atezolizumab + bevacizumab vs. sorafenib	2018	558	19.2 vs. 13.4 mo (HR 0.66; 95% CI 0.52–0.85)	6.8 vs. 4.3 mo (HR 0.65; 95% CI 0.53–0.81)
ORIENT-32 [124]	Sintilimab + bevacizumab biosimilar vs. sorafenib	2019	595	not reached vs. 10.4 mo (HR 0.57; 95% CI 0.43–0.75)	4.6 vs. 2.8 mo (HR 0.56; 95% CI 0.46–0.70)
LEAP-002 [133]	Lenvatinib + pembrolizumab vs. lenvatinib	2018	794	21.2 vs. 19.0 mo (HR 0.84; 95% CI 0.71-1.00)	8.2 vs. 8.0 mo (HR 0.87; 95% CI 0.73–1.02)
COSMIC-312 [134]	Cabozantinib + atezolizumab vs. sorafenib	2018	837	15.4 vs. 15.5 mo (HR 0.90; 95% CI 0.69–1.18)	6.8 vs. 4.2 mo (HR 0.63; 95% CI 0.44–0.91)
CARES-310 [136]	Camrelizumab + rivoceranib vs. sorafenib	2019	543	22.1 vs. 15.2 mo (HR 0.62; 95% CI 0.49–0.80)	5.6 vs. 3.7 mo (HR 0.52; 95% CI 0.41–0.65)
HIMALAYA [138]	Tremelimumab + durvalumab vs. durvalumab vs. sorafenib	2017	1324	16.4 vs. 16.6 vs. 13.8 mo (HR 0.78; 95% CI 0.65–0.93)	3.78 vs. 3.65 vs. 4.07 mo

OS, overall survival; mo, month; HR, hazard ratio; CI, confidence interval; PFS, progression-free survival

### Combination of ICIs with anti-VEGF agents

The pro-angiogenesis factors, such as VEGF, have been demonstrated to induce T-cell exhaustion by upregulating immune checkpoints, resulting in cancer immune evasion. In addition to blocking the cancer-intrinsic pathways that promote immune cells exclusion (such as Wnt-β-catenin), VEGF inhibitors or anti-angiogenesis TKIs could rebalance the immunosuppressive TME by facilitating T cell infiltration, down-regulating the immune checkpoints, decreasing the accumulation of immunosuppressive cells, as well as inducing tumor vascular normalization in HCC. These provide a rationality for the combination of VEGF inhibitors with anti-PD-1/PD-L1 therapies [123].

IMbrave150 is the first global phase III randomized trial to determine the efficacy of atezolizumab (anti-PD-L1) in combination with bevacizumab (anti-VEGF) as the first line treatment for patients with unresectable HCC [119]. Significant improvements in mOS (19.2 vs.13.4 months; HR 0.66, 95% CI 0.52–0.85; *p* = 0.0009) and mPFS (6.8 vs. 4.3 months; HR 0.65, 95% CI 0.53–0.81; *p* = 0.0001), as well as ORR according to RECIST (30% vs. 11%, *p* < 0.001) were demonstrated in the combination group compared with sorafenib monotherapy [119]. After longer follow-up, this combination maintained clinically meaningful survival benefits and had a safety profile consistent with the primary analysis [120]. So, the combination of atezolizumab with bevacizumab has become the new standard of care for patients with advanced HCC. Similarly, the ORIENT-32 phase III trial demonstrated that the PD-1 antibody sintilimab in combination with a bevacizumab biosimilar (IBI305) could improve both mOS (not reached vs. 10.4 months; HR 0.57, 95% CI 0.43–0.75; *p* < 0.0001) and mPFS (4.6 vs. 2.8 months; HR 0.56, 95% CI 0.46–0.70; *p* < 0.0001) in Chinese patients with advanced-stage HBV-associated HCC naive to systemic therapy [124]. This success is due to VEGF inhibition, which reduces VEGF-mediated immunosuppression, promotes the normalization of the tumor vasculature, and enhances the infiltration and effector function of cytotoxic T lymphocytes in microenvironment [125–127].

Very recently, in the global, open-label, phase III IMbrave050 study, among the patients at high risk of HCC recurrence following curative-intent resection or ablation, relapse-free survival (RFS) was improved in those who received atezolizumab plus bevacizumab versus active surveillance [128].

### Combination of ICIs with TKIs

TKIs including sorafenib, lenvatinib, etc., have the ability to simultaneously target VEGF, PDGF, FGF receptors, MET, and KIT. This allows them to not only inhibit angiogenesis but also exert stronger tumor-killing effects through other targets and enhance anti-tumor immunity [129]. For the past decade, sorafenib has been the only first-line systemic therapy for advanced HCC until 2018 when lenvatinib emerged as an alternative option after demonstrating non-inferiority to sorafenib [130, 131]. Currently, combinations of TKIs with ICIs are the most frequently used strategy to increase the efficacy of immunotherapy. In the LEAP-002 phase III trial, 794 patients with advanced HCC were enrolled to compare the efficacy of lenvatinib plus pembrolizumab with lenvatinib alone. Initially, in 104 patients, this combination demonstrated promising clinical activity with an ORR of 46%, mOS of 22 months, and mPFS of 9.3 months [132]. However, lenvatinib plus pembrolizumab did not meet prespecified significance for improved OS and PFS versus lenvatinib plus placebo [133]. Similar to the COSMIC-312 trial, the OS improvement did not meet the predefined statistical significance criteria even though the interim analysis revealed an improvement in PFS (HR 0.63, 95% CI 0.44–0.91; *p* = 0.0012) and a trend for a longer OS with the combination arm of atezolizumab with cabozantinib against sorafenib as the first-line therapy for unresectable HCCs [134].

In the RESCUE phase II trial, the combination of rivoceranib (or apatinib, a VEGFR2 inhibitor, TKI) with camrelizumab (SHR-1210, PD-1 antibody) showed a promising efficacy for advanced HCCs with an ORR of 34.3% [135]. In the CARES-310 phase III study of 543 HCC patients, camrelizumab plus rivoceranib showed a statistically significant and clinically meaningful benefit in mPFS (5.6 vs. 3.7 months; *p* < 0.0001) and mOS (22.1 vs. 15.2 months; *p* < 0.0001) compared with sorafenib for patients with unresectable HCC, presenting as a new and effective first-line treatment option for this population [136]. Unlike the IMbrave-150 trial, characterized by a more heterogeneous population, most of the study population in these trials is Asiatic with HBV-related HCC. Thus, further investigation is needed to better clarify the actual impact and the relevance of these results in a more assorted population, such as non-Asiatic people and non-viral HCC.

### Combination of different ICIs

The first clinical trial data came from the CheckMate 040, the combination of dual PD-1 and CTLA-4 blockades (nivolumab and ipilimumab) achieved an ORR of 31%, and a 24-month OS rate of 40% in advanced HCC who had received sorafenib before, which led to the approval of the combination as 2nd line treatment of HCCs by FDA [137]. The phase III CheckMate 9DW is undergoing (NCT04039607).

Another important progress is the combination of PD-L1 antibody durvalumab with CTLA-4 antibody tremelimumab. In the HIMALAYA phase III trial, the patients with unresectable HCC who were naïve to previous systemic treatment were randomly assigned to receive one of three study arms: tremelimumab plus durvalumab, durvalumab, or sorafenib. The primary objective of improvement in OS for tremelimumab plus durvalumab compared with sorafenib met statistical significance with a stratified HR of 0.78 (95% CI 0.66–0.92; *p* = 0.0035). The mOS was 16.4 months (95% CI 14.2–19.6) with the combination arm and 13.8 months (95% CI 12.3–16.1) with sorafenib. So, this combination was approved by the FDA for adult patients with unresectable HCC on October 21, 2022 [138, 139].

## The roles of immunotherapy-based perioperative treatment in HCC

The perioperative treatment includes adjuvant therapy, neoadjuvant therapy, and downstaging conversion therapy (i.e., convert the patients with initially unresectable HCC to resectable one). Perioperative therapy helps create surgical opportunities and reduce the risk of post-operative recurrence. Immunotherapies (particularly ICIs and their based combinations) have demonstrated to play more and more important roles, and have brought a paradigm shift in the surgical treatment of HCC [140, 141] (Table 3).

**Table 3:** The major ICI-based clinical trials in the perioperative treatment of hepatocellular carcinoma

Study	Treatment setting	Drug	Phase	*n*	Primary endpoint	Efficacy
IMbrave050 [128]	Adjuvant	Atezolizumab + bevacizumab vs. placebo	III	668	RFS	Improved RFS HR = 0.72 (95% CI 0.53–0.98; *p* = 0.012)
Checkmate 9DX [142]	Adjuvant	Nivolumab vs. placebo	III	545	RFS	NA
EMERALD-2 [143]	Adjuvant	Durvalumab ± bevacizumab vs. placebo	III	908	RFS	NA
KEYNOTE-937 [144]	Adjuvant	Pembrolizumab vs. placebo	III	950	RFS/OS	NA
JUPITER 04	Adjuvant	Toripalimab vs. placebo	II/III	402	RFS	NA
NCT04639180	Adjuvant	Camrelizumab + Rivoceranib vs. active surveillance	III	687	RFS	NA
Wang K, et al. [145]	Adjuvant	Sintilimab vs. active surveillance	II	198	RFS	27.7 vs. 15.5 mo; HR = 0.534 (95% CI 0.360–0.792; *p* = 0.002)
NCT05613478	Neoadjuvant	Camrelizumab + apatinib + TACE	III	130	RFS	NA
NCT05250843	Neoadjuvant	Sintilimab + lenvatinib + TACE/HAIC	II/III	90	RFS	NA
HCC-009 [146]	Neoadjuvant	Camrelizumab + apatinib	II	20	MPR	ORR = 16.7% 1-year RFS = 53.85%
NCT03222076 [147]	Neoadjuvant/ adjuvant	Nivolumab ± ipilimumab	II	30	AEs	mRFS = 19.53 vs. 9.4mo
NIVOLEP	Neoadjuvant	Nivolumab	II	43	RFS	NA
NCT04615143	Neoadjuvant	Tislelizumab ± levatinib	II	80	DFS	NA
NCT03867370	Neoadjuvant	Toripalimab ± levatinib	II	40	MPR	NA
NCT05056337	Conversion	Toripalimab + Lenvatinib + TACE	III	220	ORR	NA
LEN-TAC	Conversion	Camrelizumab + Lenvatinib + TACE	III	168	Conversion rate/OS	NA
NCT04042805 [151]	Conversion	Sintilimab + lenvatinib	II	36	ORR	ORR = 35% Conversion rate = 27%
NCT04843943 [152]	Conversion	Sintilimab + bevacizumab	II	30	AEs/EFS	ORR = 23.3% Conversion rate = 43%
NCT05029973 [153]	Conversion	Sintilimab + bevacizumab + HAIC	II	30	ORR	ORR = 66.7% Conversion rate = 47%

TACE, transarterial chemoembolization; HAIC, hepatic artery infusion chemotherapy; RFS, relapse-free survival; OS, overall survival; MPR, major pathological response; AE, adverse event; DFS, disease-free survival; ORR, objective response rate; EFS, event-free survival; HR, hazard ratio; CI, confidence interval; mo, month

The value of immunotherapy in adjuvant therapy has emerged, and several phase III studies are coming out and will rewrite practice in adjuvant HCC therapy. As mentioned above, in the IMbrave050 study, the combination of atezolizumab and bevacizumab significantly improved RFS in patients at high risk of HCC recurrence following curative-intent resection or ablation. This is the first phase III study of adjuvant treatment for HCC to report positive results [98]. In addition, Checkmate 9DX (nivolumab) [142], EMERALD-2 (durvalumab with or without bevacizumab) [143], KEYNOTE-937 (Pembrolizumab) [144], and JUPITER-04 (Toripalimabvs) are undergoing to evaluate ICIs for adjuvant therapy are currently underway. Very recently, in a multicenter, open-label, randomized controlled, phase II trial, adjuvant sintilimab (PD-1 antibody) significantly prolonged RFS compared to active surveillance (27.7 vs. 15.5 months; HR 0.534, 95% CI 0.360–0.792; *p* = 0.002) [145].

The neoadjuvant therapy aims to destroy the circulating tumor cells (CTCs), and microvascular invasion of occult metastasis, to decrease tumor burden, and thus decrease the probability of post-operation recurrence. The ICI-based combinations are the major trend of neoadjuvant systemic therapy. The HCC-009 phase II trial evaluated the safety and efficacy of camrelizumab plus apatinib as neoadjuvant therapy with an ORR of 16.7% and 1-year RFS rate of 53.85% in 18 resectable HCC patients [146]. Another phase II trial evaluated nivolumab +/- ipilimumab as neoadjuvant/adjuvant therapy in 27 patients with resectable HCC, and the combination therapy could significantly improve PFS compared with the nivolumab monotherapy (19.5 vs. 9.4 months, respectively). The fixed cycle of neoadjuvant therapy is usually 1.5-3 months, the reported progressive disease (PD) rates were 12-40%, and surgical delay or cancellation due to adverse events (AEs) is rare (≥ G3 AE is 17%∼23%). And neoadjuvant plus adjuvant therapy has a higher RFS than neoadjuvant therapy alone [147].

Downstaging conversion therapy aims to reduce tumor burden using locoregional or systemic therapy so that patients become amenable to surgical resection. Evidence is accumulating to suggest that it is a promising approach for improving survival in selected patients with intermediate to advanced-stage HCCs [148]. In early studies, the major approaches for conversion therapy were locoregional therapies including TACE, hepatic artery infusion chemotherapy (HAIC), transarterial radioembolization (TARE) with yttrium-90 microspheres (Y90), and radiotherapy. The downstaging rate was only 8–18% [149, 150]. Traditional systemic therapies such as chemotherapy and TKI monotherapy for HCC have relatively low response rates and therefore they are not included as part of standard management protocols of neoadjuvant or conversion therapy. However, recent advances in immunotherapy have led to a re-evaluation of the value of systemic therapy in the conversion therapy setting. A series of phase II and phase III trials have evaluated the feasibility and effectiveness of ICIs and their combinations for conversion therapy [151–153] (NCT05056337, NCT05738616). However, the expected value of conversion therapy in terms of improving survival is primarily based on small-sample cohort and retrospective studies, and further evidence is still needed to validate and optimize the conversion therapy strategy.

Some issues about downstaging conversion therapy need to be addressed: (1) How to identify specific patient subpopulations likely to benefit from this approach (the best beneficiaries)? More than 50% reduction in alpha-fetoprotein (AFP) or protein induced by vitamin K absence or antagonist-II (PIVKA-II) in 2–3 weeks after treatment can be used to determine the efficacy of conversion therapy, but still lack predictive markers for the best beneficiaries. (2) What is the best treatment strategy for conversion therapy? ORR and patterns of response are the most important factors. The combination of TKI and ICI is the most frequently used, but lack of high-level evidence of large-sample prospective study, the reported ORR (23-53%) and conversion rates (10%∼51%) and resected rate (10-43%) are various. Locoregional treatment in combination with TKI + ICI is hopeful for HCC with vascular invasion. (3) The necessity of subsequent resection for complete response (CR) patients? Subsequent resection aims to eliminate potential residual tumor cells, provide guidance for adjuvant treatment through pathological examination, and reduce drug exposure and systemic treatment-related adverse reactions (particularly for those proved to be pCR). However, pCR can be reached 30%-over 50%. There is not yet evidence indicating that subsequent resection could further prolong the survival of the CR patients. (4) The selection of adjuvant treatment after conversion therapy is based on the following criteria: the necrosis degree of the primary tumor and tumor thrombosis, R0 resection or not, pathological findings (pCR or not, microvascular invasion), alteration of tumor marker level in 1 month after surgery.

In summary, the rapid progression in immunotherapy of HCC has brought a paradigm shift in the surgical treatment of HCC. More work is needed to identify the optimal combination and to solve the drug resistance, as well as the systemic toxicity and side effects.

## The novel ways to enhance the response of HCC to ICIs

ICI immunotherapy alone still faces greater challenges such as lower response rate, or drug resistance (primary or acquired), as well as potential systemic side effects. It is urgent to understand the immunological rationale and explore novel ways to improve the efficacy of immunotherapy.

Efforts have been made to identify definitive predictive biomarkers to guide monotherapy with ICIs. Several potential biomarkers including PD-L1 expression, tumor mutational burden (TMB), microsatellite instability (MSI), and mismatch repair (MMR) have been proposed based on exploratory endpoints in HCC trials. The Wnt-β-catenin signaling pathway is activated in 30-50% of HCCs, and these tumors are also characterized by T-cell exclusion. Therefore, an activating mutation in Wnt-β-catenin signaling may be a negative predictive marker for patients with HCC treated with ICIs [154, 155]. However, no single biomarker shows powerful efficiency in predicting immunotherapeutic response [94].

Remodeling TME is another practical way to improve the efficacy of ICIs. Most HCCs are related to chronic hepatitis or liver cirrhosis by HBV or HCV infection which in turn leads to immunosuppressive status of TME and resistance to immunotherapy (“cold tumor”). So, remodeling the TME to transform the immunosuppressive state of “cold tumor” into “hot tumor” is a crucial way to improve the efficacy of immunotherapy for HCC [126, 156]. In the previous study, we have found that tumor cell-intrinsic OPN facilitates chemotactic migration, and alternative activation of macrophages, and promotes the PD-L1 expression in HCC via activation of the CSF1-CSF1R pathway in macrophages. OPN/CSF1/CSF1R axis plays a critical role in the immunosuppressive nature of the HCC microenvironment. Blocking CSF1/CSF1R prevents TAM trafficking, rebalances the shift of immunoinflammatory response shift of TME, and thereby enhances the efficacy of ICIs for the treatment of HCC [20]. IFN-α can generate a microenvironment favoring PD-1 blockade to work [157]. Mechanically, it reprograms glucose metabolism within the HCC TME, thereby liberating T-cell cytotoxic capacities and potentiating the PD-1 blockade-induced immune response. Tumor-infiltrating CD27 + CD8 + T cells may be a promising biomarker for stratifying patients for anti-PD-1 therapy [158]. Thus, the combination of IFN-α and PD-1 blockade could be a promising strategy for HCC. In addition, overexpression of MYC, which occurs in approximately 50-70% of HCCs, leads to PD-L1 overexpression and is associated with an immunosuppressive pro-tumorigenic microenvironment. Intervention of MYC activity may also be a promising approach to enhance the efficacy of immunotherapy in patients with HCC refractory to ICIs [155, 159].

The combination of TKIs has been demonstrated to remodel TME and improve the efficacy of ICIs. The effects of TKIs are not limited to VEGF signaling but also block the cancer-intrinsic pathways that promote immune cell exclusion, such as Wnt-β-catenin, MYC, or PI3K-PTEN signaling pathways [123]. We also found that lenvatinib reduced tumor PD-L1 level and Treg abundance to improve anti-PD-1 efficacy by blocking FGFR4 in HCC. Levels of FGFR4 expression and Treg infiltration in tumors could serve as biomarkers for screening patients with HCC using lenvatinib plus anti-PD-1 therapy [160]. In addition, regorafenib normalizes the vasculature in HCC, increasing the infiltration of CXCR3 + CD8 + T cells to enhance the efficacy of anti-PD-1 treatment [161].

## Emerging immunotherapies

**Fig. 2:**
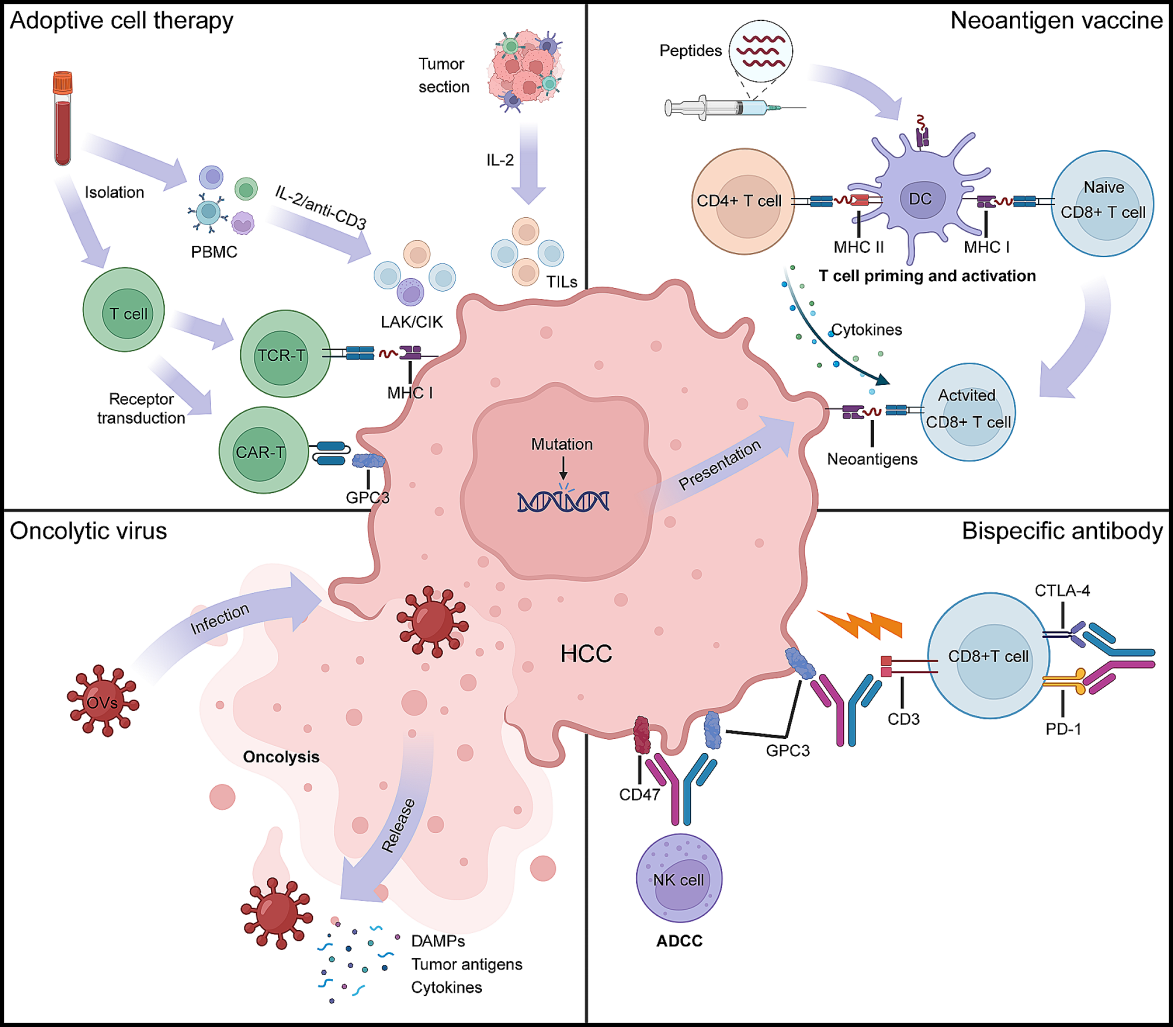
Overview of the emerging immunotherapies in hepatocellular carcinoma. Adoptive cell therapy involves the ex vivo expansion and reinfusion of immune cells, including LAK/CIK, TILs, CAR-T, and TCR-T cells, to target and eliminate tumor cells; Neoantigen vaccines can be taken by DC cells and presented to CD4 + or CD8 + T cells through MHC molecules, leading to their activation and exerting anti-tumor effects; Oncolytic viruses can directly lyse tumor cells or indirectly exert anti-tumor effects by inducing systemic immune responses; Bispecific antibodies can bridge tumor cells and T cells, enabling selective destruction of tumor cells and they can also simultaneously target two immune checkpoints on T cells to enhance immune responses. Figures were created with BioRender. Abbreviations: HCC, hepatocellular carcinoma; PBMC, peripheral blood mononuclear cell; IL, interleukin; TIL, tumor infiltrating lymphocyte; LAK, lymphokine-activated killer; CIK, cytokine-induced killer cell; MHC, major histocompatibility complex; CAR, chimeric antigen receptor; GPC3, glypican-3; DC, dendritic cell; OV, oncolytic virus; DAMP, damage-associated molecular pattern; CTLA-4, cytotoxic T-lymphocyte antigen 4; PD-1, programmed death receptor 1; ADCC, antibody-dependent cellular cytotoxicity

In addition to ICIs, many emerging strategies of immunotherapy including ACT, therapeutic cancer vaccines, and bispecific T-cell engagers (BiTE), etc. have been developed [162]. Although most of them are still in preclinical or early clinical stages, they have distinctive advantages in overcoming immune evasion, selectively killing cancer cells, and eliciting long-term immune memory (Fig. 2; Table 4).

**Table 4:** Emerging therapies for hepatocellular carcinoma

Therapies	Agents	Descriptions	Trial	Clinical results	Reference
Adoptive cell therapy	CAR-GPC3 T	Autologous T cells bearing CAR that can recognize GPC3	NCT02395250 (phase I) NCT03146234 (phase I)	PR in 2 out of 13 patients	[166]
	GPC3-7 × 19 CAR-T	CAR-GPC3 T cells secreting IL-7 and CCL19	NCT03198546 (phase I)	CR in one patient with advanced HCC	[170]
	AFP TCR-T	Autologous T Cells expressing TCRs specific for AFP	NCT03132792 (phase I)	Achieved 1 CR among 4 patients without significant liver toxicity	[164]
	HBV-specific TCR-T	Autologous T Cells expressing TCRs specific for HBV	NCT02719782 (phase I) NCT03899415 (phase I)	Confirmed the safety and tolerability of HBV-specific TCR-T cell therapy	[180, 181]
Neoantigen vaccines	Neoantigen peptides	Long peptides of predicted neoantigens derived from mutations	ChiCTR1900020990	Achieved an RFS of 7.4 months	[190]
	GNOS-PV02	Personalized DNA neoantigen vaccine	NCT04251117 (phase I/II)	Achieved 3 PR and 5 SD among 12 patients	[192]
	Neoantigen-loaded DC vaccines	DCs loaded with neoantigen peptides	NCT03067493 (phase II) NCT04912765 (phase II)	Achieved a longer DFS in patients who developed immune responses	[195]
	mRNA vaccine	Personalized mRNA neoantigen vaccine	NCT05761717	No results posted	
Oncolytic viruses	T-Vec	Engineered low-toxicity HSV expressing GM-CSF	NCT02509507 (phase Ib/II)	No fatal AE was observed	[201]
	JX-594	Genetically modified VV expressing GM-CSF and β-galactosidase	NCT01387555 (phase IIb) NCT02562755 (phase III)	Fail to provide benefits for HCC patients	[205]
	H101	Type 5 adenovirus been modified to delete the E1B55K protein	NCT05675462 (phase I)	No results posted	
Bispecific antibodies	GPC3-CD3 BsAb	Redirect T cells to HCC cells by engaging CD3 on T cells and GPC3	NCT02748837 (phase I)	No results posted	[217]
	PD-1-CTLA-4 BsAb	Synergistically target two immune checkpoints on immune cells	NCT04728321 (phase II) NCT04444167 (phase Ib/II) NCT05773105 (phase I/II) NCT05925413 (phase II)	No results posted	

CAR, chimeric antigen receptor; GPC3, glypican-3; AFP, alpha-fetoprotein; HBV, hepatitis B virus; DC, dendritic cell; T-Vec, talimogene laherparepvec; BsAb, bispecific antibody; PD-1, programmed death receptor 1; CTLA-4, cytotoxic T-lymphocyte antigen 4; IL, interleukin; CCL, C-C motif ligand; HSV, Herpes simplex virus; GM-CSF, granulocyte-macrophage colony-stimulating factor; VV, vaccinia virus; PR, partial response; CR, complete response; HCC, hepatocellular carcinoma; RFS, relapse-free survival; SD, stable disease; DFS, disease-free survival; AE, adverse event

### ACT

ACT mainly includes lymphokine-activated killer (LAK) cells, CIK cells, tumor infiltrating lymphocytes (TILs), CAR-T cells, and TCR-T cells, which involve ex vivo expansion of autologous or allogeneic immune cells and subsequent reinfusion into patients to eliminate tumor cells [4, 129, 163].

LAK cell therapy, in which the lymphocytes from peripheral blood mononuclear cells (PBMCs) were activated by IL-2 in vitro, was proposed several decades ago. However, it did not achieve significant breakthroughs in clinical efficacy [163]. Recently, gene-engineered cell therapies that can specifically target tumor-associated antigens (TAA) or tumor-specific antigens (TSA), including CAR-T and TCR-T cell therapies, are increasingly recognized as promising treatment strategies against HCC. CAR consists of three domains, including an extracellular antigen recognition domain, a transmembrane domain, and an intracellular signaling domain composed of co-stimulatory molecules. This enables CAR-T cells to specifically recognize antigens expressed on the surface of tumor cells and kill them independently of major histocompatibility complex (MHC) molecules [163]. TAAs that are often overexpressed in HCC, such as glypican-3 (GPC3), serve as excellent targets for CAR-T cell therapy [164]. GPC3 is a membrane protein that is expressed in approximately 70% of HCC cases but is rarely expressed in normal tissues, making it one of the most attractive targets in HCC. CAR-GPC3 T cell therapy has been found to inhibit the growth of GPC3-expressing HCC in xenograft and patient-derived xenograft (PDX) mouse models [165]. Two phase I clinical trials evaluated the safety of CAR-GPC3 T cell therapy and observed partial response (PR) in 2 out of 13 patients, thus providing preliminary indications of the anti-tumor effects of this therapy [166]. However, the immunosuppressive microenvironment, heterogeneity of tumor antigen expression, and off-target effects remain the challenges for the application of CAR-T therapy in HCC. Interestingly, shed GPC3, existing in a soluble form, can competitively bind to CAR-GPC3 T cells without activating them, thereby impairing their anti-tumor efficacy [167].

Many improvement strategies are explored to improve the tumor infiltration and anti-tumor properties of CAR-T cells. Soluble PD-1 antibody was expressed in CAR-GPC3 T cells to block T cell exhaustion caused by the PD-1/PD-L1 pathway [168]. IL-15 and IL-21 have been engineered into CAR-GPC3 T cells, promoting T cell expansion and survival, and enhancing their anti-tumor properties. Similarly, the expression of IL-7 and CCL19 in CAR-GPC3 T cells can promote T cell survival and tumor infiltration, leading to stronger tumor suppression compared to traditional CAR-T cells in xenograft and PDX models [169]. Intratumoral injection of CAR-GPC3 T cells secreting IL-7 and CCL19 resulted in complete tumor regression of an advanced HCC patient [170]. Recently, in a phase I trial, CAR-GPC3 T cells expressing RUNX3 (which promotes the infiltration of CD8 + T cells into tumors) were used to treat 6 HCC patients with an acceptable safety profile. Among the treated patients, one achieved PR, and 2 had stable disease (SD) [171].

Dual-target CAR-T cells have been developed to enhance the recognition ability to reduce off-target effects. CD147 is a transmembrane glycoprotein that is frequently overexpressed in aggressive HCC. CAR-T cells engineered to target both GPC3 and CD147 can selectively kill dual antigen-positive HCC cells, thereby avoiding severe on-target/off-tumor toxicity [172]. Similarly, CAR-T cells dual-targeting GPC3 and asialoglycoprotein receptor 1 (ASGR1, a liver tissue-specific protein) have been developed [173]. In addition, the feasibilities of AFP-CAR [174], NKG2D-CAR [175], and HBVs-CAR [176] T cells have also been reported.

TCR-T cells have the ability to recognize both membrane and intracellular epitopes presented by MHC, allowing them to target a wider range of tumor antigens [177]. AFP expression is predominantly intracellular or secreted, which makes AFP a more suitable target for TCR-T rather than CAR-T cell therapy [178]. In an ongoing clinical trial (NCT03132792), TCR-T therapy has already achieved 1 CR among 4 patients without significant liver toxicity [164]. HBV antigens are also promising targets for TCR-T therapy. HBV-specific TCR-T therapy has shown the potential to completely eradicate HCC. In two HCC patients who received HBV-specific TCR-T therapy, one patient showed a reduction in five of six lung metastases after one year of treatment [179]. Furthermore, two phase I clinical trials have also confirmed the safety and tolerability of HBV-specific TCR-T cell therapy [180, 181]. With the advancement of high-throughput sequencing technology, the identification of neoantigen-specific T cells has become possible. This brings new possibilities for the development of neoantigen-specific TCR-T cell therapy, making it a promising direction for future research. Dominant ENTPD6 neoantigen has been demonstrated strong immunogenicity. TCRs that specifically recognize this epitope have been identified and engineered into TCR-T cells. These neoantigen-specific TCR-T cells have shown anti-tumor effects in mouse models [182].

### Neoantigen vaccines

Traditional therapeutic cancer vaccines were typically based on tumor lysates or TAAs such as AFP and GPC3. However, these attempts were largely unsuccessful in producing significant clinical efficacy due to the weak immunogenicity and low specificity of tumor lysate antigens and TAAs. Thus, enhancing immunogenicity and specificity is crucial for the development of cancer vaccines [183]. Neoantigens refer to epitopes derived from genetic mutations, alternative splicing, or post-translational modifications that can be presented by the MHC molecules on the surface of tumor cells and recognized by T cells as neoepitopes [184]. Neoantigens are ‘foreigners’ to the body, thereby circumventing central tolerance of self-epitopes. Moreover, neoantigens are exclusively expressed by tumor cells, avoiding off-target damage to normal tissues. These advantages make neoantigen vaccines the most attractive therapeutic approach in the field of cancer vaccination. The candidate neoantigens are delivered to the patient’s body in the format of DNA vaccines, peptides, DC vaccines, or mRNA vaccines [183, 185].

HCC is a tumor with a moderate TMB, with approximately 2 mutations per megabase [186, 187]. Several preclinical studies have identified the neoantigen repertoire derived from these mutations and demonstrated that they could be potential treatment targets of HCC [182, 188, 189]. However, in a clinical study, the neoantigen vaccine treatment did not significantly improve the prognosis of HCC after the operation, even though 2 out of 10 patients remained relapse-free, achieving an RFS of 7.4 months at the end of the trial [190]. These indicate that more barriers need to be overcome for neoantigen vaccines to generate clinically effective tumor immunity in HCC.

The most readily conceivable strategy is to use ICIs to counteract the immunosuppressive microenvironment in HCC and enhance the efficacy of neoantigen vaccines. The combination of neoantigen peptides with anti-PD-1 treatment resulted in a potent anti-tumor immune response in HCC mouse models, leading to tumor regression in 80% of cases and establishing durable immune memory [184, 191]. A clinical trial investigating the combination of personalized neoantigen vaccine GNOS-PV02 and pembrolizumab for advanced HCC is currently underway (NCT04251117). Preliminary results demonstrated that among the initial 12 patients, 3 achieved PR, and 5 had SD [192].

Another strategy is to enhance the uptake and presentation of neoantigen vaccines by APCs. Leveraging red blood cells to deliver neoantigen vaccine-encapsulating polymeric nanoparticles can result in their preferential accumulation in the spleen, thereby enhancing their presentation by APCs [193]. Utilizing a virus-like silicon vaccine [194] or nano-vaccine [48] to co-deliver neoantigens and Toll-like receptor 9 (TLR9) agonists to DCs can activate the maturation of DCs, thereby enhancing antigen presentation and facilitating robust CD8 + T cell responses. Neoantigen peptides can also be directly loaded onto DCs to create DC vaccines, but it is still a challenge to induce a large number of antitumor T cells in a short period of time and their efficacy might be limited due to T cell exhaustion. In a phase II clinical trial, the combination of neoantigen-loaded DC vaccines and ACT not only provided an immediate supply of tumor-specific T cells to HCC patients but also induced long-term immune memory, thereby achieving complementary and synergistic effects. The patients who developed immune responses achieved a longer DFS [195].

Another obstacle for neoantigen vaccines is the intrinsic nature of HCC. The average number of somatic mutations per HCC patient is around 70, and about 30% of the mutations are predicted by algorithms to generate candidate neoantigens [190]. However, only 0.6-2% of the candidate neoantigens can be recognized by autologous T cells. Multi-omics approach has been used to discover the exome-derived neoantigens. Unfortunately, HLA peptidomics is unable to identify any exome-derived mutated HLA ligands in HCC [189]. These indicate that truly effective neoantigen peptides are very rare in the neoantigen vaccines produced with current technologies. Some strategies, such as the combination with radiotherapy to enhance the transcription of pre-existing neoantigens and their presentation on the tumor membrane [196], interfering with MMR genes [197] or RNA splicing [198] to increase the number of neoantigens, and improving the detection capability of neoantigens as well as the accuracy of prediction algorithms, etc., might be hopeful in overcoming this problem.

### OVs

OVs possess the unique ability to selectively replicate within tumor cells and induce their death while sparing normal cells unaffected. In addition to directly lysing tumor cells, OVs can also exert anti-tumor effects by indirectly inducing systemic immune responses. Following oncolytic tumor cell death, the release of cell damage-associated molecular patterns (DAMPs), tumor antigens, and cytokines such as type I interferons and TNF-α activates innate immune responses and tumor-specific adaptive immune responses to further eliminate tumor cells [199].

Talimogene laherparepvec (T-VEC) is an engineered herpes simplex virus 1 (HSV-1) that was modified to attenuate neurotoxicity and incorporate the expression of GM-CSF to promote DC maturation. Due to its excellent performance in clinical trials for the treatment of metastatic melanoma, T-VEC became the first widely approved OV product, marking a milestone in oncolytic virotherapy [200]. A multicenter phase Ib/II clinical trial has shown early safety and feasibility of T-Vec combined with pembrolizumab in patients with HCC and liver metastases [201].

Pexastimogene devacirepvec (JX-594), a genetically modified vaccinia virus (VV), was approved as an orphan drug for the treatment of HCC in 2013 [202]. The viral thymidine kinase (TK) gene was deleted in JX-594 for its selective replication within TK-expressing tumors and GM-CSF as well as β-galactosidase was expressed to activate the immune response. In addition, JX-594 can also selectively infect and disrupt tumor-associated vascular endothelial cells to reduce tumor blood supply, leading to tumor necrosis [203]. In a phase I (NCT00629759) and a subsequent phase II (NCT00554372) clinical trial, intrahepatic injection of JX-594 showed positive therapeutic efficacy in HCC, with a dose-dependent benefit to OS [204]. However, in the phase IIb trial, JX-594 did not provide benefit for advanced HCC patients as a second-line treatment after sorafenib failure [205], or in combination with sorafenib (Phase III, NCT02562755). The combination of JX-594 with nivolumab achieved an OR of 33.3% as a first-line therapy (NCT03071094), however the study was terminated due to the significant risk of severe adverse events, as well as the failures of JX-594 and Nivolumab in their respective pivotal trials (PHOCUS and CheckMate 459).

Oncorine (H101) is a type 5 adenovirus that has been modified to delete the E1B55K protein, enabling it to selectively replicate in tumor cells with defective P53 function [206]. In 2005, H101 was approved in China for the treatment of nasopharyngeal carcinoma in combination with cisplatin and 5-fluorouracil [207]. Intraperitoneal injection of H101 has shown promising outcomes in the treatment of malignant ascites caused by various cancers including HCC, and it has also induced tumor-specific immune responses [208]. In a retrospective study of 476 HCC patients, the combination of H101 with TACE significantly prolonged OS and decreased tumor-specific mortality [209]. A clinical trial is currently ongoing to investigate the safety and efficacy of H101 combined with tislelizumab plus lenvatinib in HCCs as a second-line treatment (NCT05675462).

Recently, a series of preclinical studies have explored the feasibility and potential of various improved oncolytic virotherapy approaches in HCC. Morreton Virus (MORV), a novel oncolytic Vesiculovirus, did not induce serious neurological adverse events compared with well-studied vesicular stomatitis virus (VSV) and had a potent antitumor efficacy in HCC xenograft mouse models [210]. Our research group has recently developed a fourth-generation oncolytic adenoviruses product named OncoViron. Several modifications have been made to OncoViron to enhance its specificity and efficacy, including the fusion of the oxygen-dependent degradation (ODD) domain of HIF-1α with the E1a protein to restrict its replication only in the hypoxic TME, silencing the E1B55K protein to ensure its selective replication in TP53-inactivated tumors, the triple chimerization of three serotype adenoviruses with enhanced infectivity and viability, and the incorporated expression of IL-12, IFN-γ, and CCL5 in OncoViron to enhance the anti-tumor immune response. These modifications endowed OncoViron with potent capabilities against various types of solid tumors including HCC, and the ability to synergize with anti-PD-1 or CAR-T therapies [211].

### Bispecific antibodies (BsAbs)

BsAbs possess the ability to simultaneously bind to different epitopes, enabling dual specificity. Based on their molecular engagements, BsAbs can be categorized into two major types: trans co-engagement, which bridges two cell types, such as EpCAM-CD3 BsAbs that redirect T cells to selectively destroy cancer cells; and cis co-engagement, which engages two molecules on the same cell membrane, such as PD-1-CTLA4 BsAbs that simultaneously target both immune checkpoint molecules to synergistically enhance immune responses [212].

Bridging two cell types is the most unique function of BsAbs, which is commonly utilized to bring immune effector cells in close proximity to tumor cells, thus reducing systemic toxicity and enhancing therapeutic efficacy [213]. EpCAM-CD3 BsAb was the first BiTE tested in HCC [214]. Catumaxomab, an EpCAM-targeting antibody has been approved for the treatment of malignant ascites in Europe [215]. GPC3 is another attractive target for BsAbs in HCC. GPC3-CD3 BsAb induced robust tumor regression in xenograft mouse models of HCC [216]. A phase I trial to evaluate the safety and efficacy of GPC3-CD3 BsAb has been completed [217]. The co-engagement of the inhibitory immune checkpoint CD47 with GPC3 to generate a GPC3-CD47 BsAb which could enhance the Fc-mediated effector functions against HCC through both neutrophil and macrophage-dependent mechanisms [218]. Viral antigens expressed on HBV-positive HCC cells can also serve as targets for BsAbs [219]. Two designed BsAbs simultaneously targeting HBV envelope proteins (HBVenv) and CD3 or CD28 synergistically eliminated HCC cells through both cytotoxic and cytokine-mediated mechanisms [220].

Dual blockading the immune checkpoints to enhance T cell responses is another strategy for designing BsAbs. The BsAb targeting PD-1 and CTLA4 has been shown to promote CD8 + PD-1^int^ TILs expansion and mediate regression of HCC [221]. PD-1-CTLA-4 BsAb MEDI5752 demonstrated even more potent immune activation and lower toxicity compared with the conventional combination of monoclonal antibodies [222]. Thus, a series of clinical trials evaluating the efficacy and safety of PD-1-CTLA-4 BsAb as monotherapy (NCT04728321) or in combination with lenvatinib (NCT04444167), regorafenib (NCT05773105) and TACE (NCT05925413) are currently underway for the treatment of advanced HCC.

### Future prospectives

Immunotherapy has brought new hope and a paradigm shift in the treatment of HCC. However, due to the complex microenvironment of both the liver (chronic liver disease background) and HCCs, ICI immunotherapy still faces great challenges such as lower response rate, or drug resistance (primary or acquired), as well as potential systemic side effects. Many factors contributed to the resistance to ICI therapy, which includes the T cells exclusion and dysfunction in the TME, defects in antigen processing and lack of tumor-associated antigens, presence of alternative inhibitory immune checkpoints (such as VISTA, TIM-3, LAG-3, and TIGIT), as well as the tumor-cell extrinsic factors such as immunosuppressive cells (Tregs, TAMs) and inhibitory cytokines (TGF-β) (so-called “cold” tumors). Efforts to overcome the resistance to ICI therapy have been focused on overcoming T cell-related deficiencies and/or suppressing immunosuppressive populations in the TME, enhancing antigen presentation, and improving recognition of effector immune cells. Combination (ICIs with other treatment strategies or immunotherapies themselves) is a practical way to enhance the efficacy of immunotherapy. In the future, more efforts are needed to identify the optimal combination, as well as solve the systemic side effects. Repolarization of the immunosuppressive microenvironment is also a hopeful way. Furthermore, in-depth exploring the factors contributing to the “cold” tumors, and exploring effective ways to overcome and transform them into “hot” ones will enhance the effectiveness of ICIs in HCC. More clinical evidence is urgent to support the clinical applications of emerging novel immunotherapies such as ACT, personalized neoantigen vaccines, OVs, and BsAbs, as well as their combinations with ICIs.

## Data Availability

No datasets were generated or analysed during the current study.

## Abbreviations

HCC: Hepatocellular carcinoma
ICI: Immune-checkpoint inhibitor
ACT: Adoptive cell therapy
TME: Tumor microenvironment
HBV: Hepatitis B virus
HCV: Hepatitis C virus
PD-1: Programmed death receptor 1
DC: Dendritic cell
TAM: Tumor-associated macrophage
IL: Interleukin
M-CSF: Macrophage colony-stimulating factor
TGF-β: Transforming growth factor-beta
TNF-α: Tumor necrosis factor-α
PD-L1: Programmed death-ligand 1
TIM-3: T cell immunoglobulin and mucin domain-3
Treg: Regulatory T cells
Siglec-10: Sialic-acid-binding Ig-like lectin 10
S100A9: S100 calcium-binding protein A9
IGF: Insulin-like growth factor
CCL: C-C motif ligand
OPN: Osteopontin
CSF1: Colony stimulating factor 1
CSF1R: Colony stimulating factor 1 receptor
scRNA-seq: Single-cell RNA sequencing
MDSC: Myeloid-derived suppressor cell
CCR: C-C chemokine receptor
TAN: Tumor associated neutrophil
PMN-MDSC: Polymorphonuclear myeloid-derived suppressor cell
GM-CSF: Granulocyte-macrophage colony-stimulating factor
PGE2: Prostaglandin E2
NLR: Neutrophil-lymphocyte ratio
NET: Neutrophil extracellular trap
APC: Antigen-presenting cell
cDC: Conventional dendritic cell
pDC: Plasmacytoid dendritic cell
TEX: Exhaustion T cells
mregDCs: Mature dendritic cells enriched in immunoregulatory molecules
Th: Helper T cell
CIK: Cytokine-induced killer cell
ILC: Innate lymphoid cell
NK: Natural killer
IFN: Interferon
TEM: Effector memory T
NAD+: Nicotinamide adenine dinucleotide
NMN: Nicotinamide mononucleotide
ADCC: Antibody-dependent cellular cytotoxicity
CAR: Chimeric antigen receptor
ICOS: Inducible T-cell co-stimulator
ICOSL: Inducible T-cell co-stimulator ligand
NCR: Natural cytotoxicity-triggering receptor
TOX: Thymocyte selection associated high mobility group box
CTLA-4: Cytotoxic T-lymphocyte antigen 4
LAG3: Lymphocyte activation gene 3
TRM: Tissue-resident memory T cell
Tfh: T follicular helper cell
TIB: Tumor-infiltrating B cell
PC: Plasma cell
TLS: Tertiary lymphoid structures
NASH: Non-alcoholic steatohepatitis
Breg: Regulatory B cell
TET2: Ten-eleven translocation-2
CAF: Cancer-associated fibroblast
ECM: Extracellular matrix
HSC: Hepatic stellate cell
MIF: Migration inhibitory factor
CXCL: C-X-C motif ligand
CLCF1: Cardiotrophin-like cytokine factor 1
DDR1: Discoidin domain receptor 1
EC: Endothelial cell
LSEC: Liver sinusoidal endothelial cell
VEGF: Vascular endothelial growth factor
TACE: Transarterial chemoembolization
RFA: Radiofrequency ablation
TKI: Tyrosine kinase inhibitor
OV: Oncolytic virus
ORR: Objective response rate
FDA: U.S. Food and Drug Administration
CR: Complete response
OS: Overall survival
PFS: Progression-free survival
HR: Hazard ratio
CI: Confidence interval
RFS: Relapse-free survival
PD: Progressive disease
AE: Adverse event
HAIC: Hepatic artery infusion chemotherapy
TARE: Transarterial radioembolization
Y90: Yttrium-90 microspheres
AFP: Alpha-fetoprotein
PIVKA-II: Protein induced by vitamin K absence or antagonist-II
CR: Complete response
TMB: Tumor mutational burden
MSI: Microsatellite instability
MMR: Mismatch repair
BiTE: Bispecific T-cell engager
LAK: Lymphokine-activated killer
TIL: Tumor infiltrating lymphocyte
PBMC: Peripheral blood mononuclear cell
TAA: Tumor-associated antigen
TSA: Tumor-specific antigen
MHC: Major histocompatibility complex
GPC3: Glypican-3
PDX: Patient-derived xenograft
PR: Partial response
SD: Stable disease
ASGR1: Asialoglycoprotein receptor 1
TLR: Toll-like receptor
DFS: Disease-free survival
DAMP: Damage-associated molecular pattern
T-VEC: Talimogene laherparepvec
HSV-1: Herpes simplex virus 1
VV: Vaccinia virus
TK: Thymidine kinase
MORV: Morreton Virus
VSV: Vesicular stomatitis virus
ODD: Oxygen-dependent degradation
BsAb: Bispecific antibody
HBVenv: HBV envelope proteins
